# Should we consider the infusion of lipid emulsion in the resuscitation of poisoned patients?

**DOI:** 10.1186/s13054-014-0457-5

**Published:** 2014-07-30

**Authors:** Grant Cave, Martyn G Harvey

**Affiliations:** Department of Critical Care, Tamworth Rural Referral Hospital, Tamworth, NSW 2340 Australia; Waikato Hospital, Pembroke St, Hamilton, 3204 New Zealand

## Abstract

The use of intravenous lipid emulsions (ILEs) as antidote in local anaesthetic systemic toxicity has gained widespread support following convincing data from animal models, and successful case reports in humans. Proposed beneficial mechanisms of action for ILEs include intravascular sequestration of intoxicant and subsequent enhanced redistribution to biologically inert tissues, augmentation of fatty acid utilisation for ATP synthesis in the context of metabolic poisoning, and direct cardiotonic and ion channel effects. The evidence base for use of ILEs in acute drug intoxication is evolving. The present evidence supports use of ILEs only in local anaesthetic systemic toxicity and in lipophilic cardiotoxin intoxication when there is an immediate threat to life, and other therapies have proven ineffective.

## Introduction

Intravenous lipid emulsion (ILE) has a long history of use as a parenteral nutrition formulation in both adult and paediatric patients. Over the past decade, ILE also emerged as a novel treatment for iatrogenic and intentional drug poisonings. Progress from nutritional replacement to antidote has been rapid, necessitating regular review of the rationale and evidence base for administration.

As early as 1998 Weinberg and colleagues reported attenuation of bupivacaine toxicity in a rat model [1], following a chance finding while attempting to replicate local anaesthetic effects in disorders of carnitine metabolism. These findings were subsequently confirmed in intact dogs [2]. Still earlier findings of lipid modulation of free drug concentrations have been reported as far back as 1974 [3]. Promising laboratory studies such as these may have forever lacked clinical impact but for two sentinel cases of ILE use in human poisoning.

In 2006 Rosenblatt and colleagues [4] reported successful resuscitation with ILE following bupivacaine and mepivacaine-induced cardiac arrest in a middle aged man undergoing brachial plexus block. Prior treatment with advanced cardiac life support, defibrillation, and multiple doses of vasopressor and antiarryhthmic medications had failed to effect return of spontaneous circulation. Normal sinus rhythm was nevertheless restored following one 100 ml bolus of ILE with rapid return of haemodynamic stability. The patient was discharged neurologically intact.

Likewise in 2007, Sirianni and colleagues [5] published a case of overwhelming bupropion and lamotrigine self poisoning in a 17 year old girl. Cardiovascular collapse refractory to standard resuscitative measures persisted for 70 minutes before administration of 100 ml ILE. Immediately after injection the patient exhibited a sustained stable circulation and survived to hospital discharge. These, the first case reports of successful ILE use in local anaesthetic systemic toxicity (LAST) and non-local anaesthetic intoxication, respectively, are unique for a number of reasons. In both instances attending physicians were presented with desperate and apparently irretrievable clinical scenarios secondary to poisoning. The decision to employ ILE was made late in the course of resuscitative attempts as a ‘last gasp’ measure, with only limited animal data to support utilisation. And, in both circumstances, use was associated with dramatic success and patient survival.

The dissemination of these and other subsequent cases of clinical use of ILE has seen ILE make the transition from bench-top modelling to clinically utilised antidote without the evidence base commonly associated with introduction of newly formulated drugs. There have been many recent systematic reviews on this topic of a high standard [6-10]. It is the purpose of the present narrative review to examine recent literature with a particular focus on advances in understanding of the mechanism of ILE, and to evaluate the evidence supporting ILE administration, and thereby establish whether any changes to present recommendations for utilisation are warranted. PubMed was searched for the 5 years since the authors previous review [6], using the search terms intravenous AND fat emulsion OR lipid emulsion OR intralipid AND "toxicity" OR "resuscitation" OR "rescue" OR "arrest" OR "antidote" and a filter for relevance. Only English language papers were included.

## Rationale for use of intravenous lipid emulsion in toxicology

There are presently four small human trials involving the use of ILE: one clinical randomised controlled trial of limited methodologic quality, one case–control study and two pharmacokinetic evaluations. While this small body of human trial work is of importance, it is not sufficient to form a view on the use of ILE in toxicology. The weight of present data is in benchtop and animal models and collated human case reports. While it is possible to evaluate such an evidence base, there are clear limits to the breadth such evidence can generalise to everyday clinical practice.

In the setting of immediate threat to life where other treatments are not proving effective, it is reasonable to use an agent when there is an established rationale for use and 'all or nothing' cases such as those described above have been reported. Table 1 lists agents for which the criterion of positive benchtop evidence and reported clinical use associated with a positive outcome has been fulfilled.

**Table 1:** **Agents for which there is both positive benchtop evidence for class effect and reported clinical use associated with a positive outcome**

Na channel antagonists	
Local anaesthetics [11-14]	
Tricyclic antidepressants [9,15-24]	
Doxepin	
Imipramine	
Amitriptyline	
Dothiepin	
Flecainide (benchtop model evidence equivocal)	
Propafenone	
Cocaine	
Ca channel blockers [9,25-27]	
Verapamil	
Diltiazem	
Beta blockers [9,28,29]	
Propanolol	
Carvedilol	
Nebivolol	
Miscellaneous [30,31]	
Haloperidol	

Given the above rationale, this paper first outlines the available laboratory evidence on mechanism of action of ILE as antidote, and then progresses to review reported clinical experience.

## Pharmacologic mechanism of action for intravenous lipid emulsion

Knowledge of the proposed pharmacologic mechanisms of action of ILE in acute intoxication is important when considering clinical application. Despite extensive and ongoing study the precise mode of action for ILE in many poisonings remains to be fully elucidated. At present both pharmacokinetic and pharmacodynamic actions have been postulated to play a role in the action of ILE as antidote. Whilst synergy between proposed actions may exist, the relative contributions of each remain uncertain. It is quite possible that each mechanism may variably contribute to clinically observed improvement depending on unique combinations of patient factors, the mode and duration since poisoning, and the particular intoxicant.

### Pharmacokinetic modulation/lipid sink theory

First forwarded by Weinberg in 1998 [1], this hypothesis proposes that, following ILE injection, lipophilic intoxicant preferentially distributes into the newly created intravascular lipid compartment, and is held away from the site of toxic action. The hypothesis has been extended to include effects on subsequent enhanced redistribution of lipophilic intoxicants, with increased blood carriage thought to speed transport of toxin from target organ to peripheral adipose tissue depots. This so called ‘lipid sink’ thesis has served to guide much of the early development of ILE therapy, with the majority of agents studied thus far exhibiting high lipid solubility.

Evidence in support of the lipid sink has been derived from both pre-clinical models and human reports. Following their study with the prototypical lipophilic local anaesthetic bupivacaine in whole rat models of cardiotoxicity [1], Weinberg and colleagues subsequently demonstrated enhanced myocardial washout of bupivacaine when cardiac perfusate was spiked with ILE in an isolated heart model [11]. Both greater myocardial bupivacaine loss (*P* < 0.0016) and increased coronary sinus effluent bupivacaine concentration (*P* < 0.008) were recorded, in keeping with rapid tissue detoxification. Similarly, Chen and colleagues [12] have shown lesser myocardial bupivacaine concentrations, and associated dose-dependent increases in rate-pressure product for isolated rat hearts subjected to lipid emulsion.

Subsequent authors investigating the effect of ILE injection in whole rats have demonstrated evidence of enhanced drug redistribution with reduced bupivacaine concentration in heart, brain, lung, kidney and splenic tissue observed following ILE treatment [13]. The hepatic bupivacaine concentration was increased. In this model ILE use was associated with greater bupivacaine clearance, and prolonged half-life (T1/2) compared with control animals, in keeping with favourable modulation to bupivacaine pharmacokinetics.

Support for a lipid sink effect is not confined to lipophilic local anaesthetics, with animal models and human cases demonstrating an effect with a range of disparate intoxicants, including calcium channel blockers [25-27], lipophilic beta-blockers [28,29], tricyclic antidepressants [15-17], antipsychotics [30,31], and antiarrhythmics [32]. Despite lacking commonality in their toxicodynamic mechanisms, agents demonstrating response to ILE treatment typically exhibit high lipid solubility (log D (octanol:water partition coefficient at physiologic pH) >2; bupivacaine logD = 3.65), consistent with the proposed pharmacokinetic mechanism. Indeed, drug partitioning constants and volumes of distribution have been investigated as predictors of ILE responsiveness. French and co-workers in 2011 [33] demonstrated *in vitro* sequestration of lipophilic drugs in human plasma spiked with lipid emulsion, and subsequently assembled a compendium of predicted lipid extraction efficiency on the basis of the intrinsic pharmacologic parameters for over 50 commonly used drugs.

*In vivo* studies using non-local anaesthetic drugs have confirmed sink effects in intact animal models and human subjects. In a rabbit model of intravenous clomipramine toxicity, increased total blood concentrations of clomipramine were seen in concert with improved blood pressure following ILE injection, consistent with sequestration of toxin into an intravascular lipid phase [34]. Litonius and colleagues [35] similarly demonstrated an increased total amitriptyline concentration with a decrease in free amitriptyline fraction in amitriptyline toxic pigs when ILE was given after amitriptyline infusion. Notably, however, there was no improvement seen in haemodynamic metrics nor survival in this model. The same group furthermore demonstrated that pretreatment with ILE improved haemodynamics during an amiodarone infusion, and resulted in increased amiodarone total concentration and decreased free drug concentration [32]. ILE has also been shown to reduce brain concentrations of amitriptyline when given following intravenous infusion and tissue distribution time in swine [36]. In the same experiment a non-statistically significant reduction in cardiac amitriptyline concentrations was additionally reported (*P* = 0.07).

Human pharmacokinetic data are limited to two clinical studies and sporadic case reports. Minton and colleagues [37] demonstrated a statistically non-significant increase in plasma amitriptyline concentrations in subjects in pharmacokinetic steady state subjected to infused lipid emulsion. Free drug concentrations were not measured. Litonius and colleagues [38] furthermore found a decreased half-life for a nontoxic dose of bupivacaine in blood after administration of ILE in eight normal subjects, consistent with an effect on tissue redistribution. A decrease in free verapamil concentration was reported after treatment with ILE for intoxication [39], commensurate with clinical improvement. In another report, increased total blood amitriptyline concentrations were seen following ILE therapy in a case of mixed overdose with prominent tricyclic toxidrome [40].

While attractive in its simplicity, emerging evidence suggests application of the sink theory must be approached with some caution in all but a few overdose scenarios. A pure sink effect may be important in cardiac arrest due to direct intravenous bolus administration of intoxicant - the centralised circulation meaning that rapid equilibration of heart and brain concentrations with the introduced, centrally confined lipid phase may be possible. Subsequent redistribution of intoxicant to peripheral fat depots on return of effective circulation would conceivably ensue - such a situation could be hypothesized to occur in most forms of LAST.

Any pharmacokinetic effect for ILE in the non-arrested patient likely depends on both the pharmacokinetic phase and circulatory status at the time of intoxication. Increased plasma carriage for lipophilic toxins with ILE injection will increase drug transport between organ systems according to a complex interplay of tissue drug affinity, relative concentration, and perfusion. As such, net movement of intoxicant may not prove universally beneficial (that is, from target organ to peripheral depot). For example, in one study investigating the effect of ILE on thiopental anaesthesia in rabbits, early lipid administration appeared to increase the depth, but not overall duration, of anaesthesia. This could be explained by the action of lipid emulsion as a high affinity conduit - maintaining blood concentrations able to interact with the brain during early redistribution to hydrophilic tissues, before augmenting later redistribution from rapidly perfused to lesser perfused lipophilic tissues [41].

Perhaps most vexing is the issue of ILE administration following enteric overdose. Data suggesting that ILE injection may actually augment absorption of lipophilic toxins from the gastrointestinal lumen have emerged, with increased mortality reported when ILE was administered early in the course of oral amitriptyline and verapamil toxicity [42], and earlier death following ILE in rectal thiopentone overdose [43] in rodent models. Documented increases in plasma intoxicant concentrations were reported in both studies. Effects on enhanced gastrointestinal uptake are likely to be limited to the absorptive phase of intoxication. For example, when ILE was administered 5 minutes after oral poisoning with the lipophilic organophosphate parathion in rats, no difference was observed in time to respiratory arrest. When given at 20 minutes (at the time of anticipated peak intravenous parathion concentration), ILE effected a significant increase in survival times, consistent with beneficial augmentation of drug redistribution [44].

The sum of what is known on the pharmacokinetic effects of ILE as antidote seems to be that there are demonstrable effects on absorption, distribution and redistribution for lipophilic intoxicants. Early ILE use in enteric poisoning may be contraindicated because of potential for enhancing gastrointestinal absorption, albeit any such adverse effect is likely dependent on adequate intestinal perfusion, and mitigated somewhat by development of shock-like states secondary to intoxication. The clinical corollary of this would be that effects on blood concentrations following ILE administration likely depend on the timing of administration relative to both the pharmacokinetic phase and the mode of intoxication. It would also seem prudent to ensure actions necessary to mitigate gastrointestinal absorption, such as activated charcoal, were taken before or contemporaneous to lipid emulsion.

### Direct cardiotonic effect

While clinical and experimental effects spanning agents and organ systems favour a pharmacokinetic mechanism as important in the action of ILE, they are not a complete explanation. Recent data have emerged that suggest lipid emulsions at the doses used have direct cardiotonic effects. Experimentally, rats infused with lipid emulsion showed increased blood pressure and aortic flow rates relative to the same volume of saline. Additionally, isolated rat hearts in isovolumetric contraction were seen to contract more forcefully, with increased oxygen demand, when perfusate was augmented with lipid emulsion [45]. Stehr and colleagues [46] additionally demonstrated a positive effect on bupivacaine toxic cardiac myocytes at levels below those that could have effected any 'sink' phenomenon. The same investigators furthermore modelled the relative contributions of volume, sink and cardiotonic effects in an *in silico* model for the observed effect on recovery of rats from bupivacaine-induced shock. The model of best fit included all three factors, with the direct cardiotonic effect being the most prominent factor responsible for observed improvement [47].

It seems likely that direct cardiotonic effects play a significant role as one of the mechanisms responsible for resuscitation from drug-induced cardiotoxicity. This may be particularly so in the arrested or critically compromised circulation in order to offer immediate support before any distributive effects are likely to occur.

### Fatty acid metabolism theory

There is evidence that metabolic pathways influenced by lipid emulsion are important in the experimental mechanism of action of ILE. Partownavid and colleagues [48] demonstrated failure of ILE rescue in rat bupivacaine toxicity in the presence of the inhibitor of fatty acid metabolism CTV. A dose-dependent relationship for CTV on both resuscitation outcome and measures of cardiac function post-resuscitation was additionally observed. In an associated laboratory experiment, bupivacaine toxic mitochondria were more resistant to external calcium pulses, thought to be a mechanism effecting cell death, when treated with ILE prior to extraction. Conversely, Bania and colleagues [49] reported no adverse effect for oxfenicine, a blocker of fatty acid transport into mitochondria, on survival time during verapamil infusion in whole rats.

While experimental data showing reduction in myocardial bupivacaine concentration post-ILE demonstrate that pharmacokinetic mechanisms play a role in ILE resuscitation, these metabolic experiments are compelling that metabolic and cardiotonic considerations are at least a necessary, if not sufficient, mechanism of action in experimental bupivacaine toxicity. This, in concert with the data on direct cardiotonic effects, is particularly important in the clinical development of ILE as antidote, as it suggests that pharmacokinetic testing alone will tend to incompletely evaluate the potential for antidotal action of ILE. Metabolic factors may furthermore hold differential importance depending on the toxic compound.

### Ion channel modulation theory

Free fatty acids, the levels of which increase with ILE, are known to have effects on both sodium and calcium ion channel function. Arachidonic, linoleic and linolenic acid all increased calcium currents through activated calcium channels in isolated guinea pig cardiac myocytes [50]. This effect appears to be directly on the channel, not modulated by any cellular second messenger system. Such an effect could be particularly important in ameliorating toxicity from calcium channel blockers, and could potentially be responsible for the short, direct cardiotonic effect seen with ILE.

Mottram and colleagues [51] demonstrated that stearic acid partially antagonises cultured human voltage-gated sodium channels. This antagonist effect was shown experimentally to partially reverse the sodium channel antagonism seen with bupivacaine. Nadrowitz and colleagues [52] have also demonstrated partial reversal of bupivacaine-induced sodium channel antagonism on the Nav15 sodium channel, known for particular tropism with bupivacaine, with lipid application in cultured human kidney cells. Notably, in cases where lipid emulsion has been reported as having a positive outcome as an antidote, sodium channel antagonists are prominent intoxicants [18-22], and very rapid resolution of QRS duration is often reported.

## Clinical use

### Human trials

Few investigators have studied ILE in human populations. Taftachi and colleagues [53] reported the use of a lipid emulsion preparation in a randomised trial evaluating the effect on conscious state in drug-induced coma. In addition to standard supportive care, 15 patients were given an infusion of 10 ml/kg 10 % lipid emulsion. A statistically significant increase in Glasgow Coma Scale (GCS) of 2 points was seen in the ILE group. No reported differences were seen for rates of intubation (the primary powered endpoint) or time to extubation. This study has a number of methodological flaws limiting generalisability, including absence of a control solution, and no blinding in acquisition of key study metrics (GCS). Additionally, the study tested a number of endpoints without statistical correction for repeat analysis. Perhaps the greatest contribution of this work is in demonstrating that trials involving ILE in toxicology are feasible.

Gil and colleagues [54] evaluated the effect of ILE on glyphosate intoxication. After a positive case report, their institution began treating glyphosate intoxication with ILE along with supportive care from January 2012. A two-tiered approach to ILE administration was taken, with those ingesting less than 100 ml of herbicide thought to be at lesser risk of toxicity and given 20 ml/hour of 20 % lipid emulsion, and those ingesting more than 100 ml of herbicide treated with a load of 500 ml of 20 % lipid emulsion over 2 to 3 hours and then maintained on 42 ml/hour for the next 24 hours. Historical controls were taken from the previous 2 years of practice, matched for volume and timing of ingestion. Twenty-two cases and controls were compared, with 13 higher dose intoxicant patients in each group. No hypotension or arrhythmia was seen in the ILE-treated group versus rates of 40.9 % (hypotension) and 22.7 % (arrhythmia) in the control group. These differences reached statistical significance. The preponderance of hypotension and arrhythmia was seen in controls who ingested higher doses of intoxicant. Additionally, death (one case) and acute kidney injury (three cases) were seen only in the control group, and the rate of respiratory failure was lower in the ILE group (14 % versus 32 %), although these differences did not reach statistical significance. It is considered most likely that surfactants associated with the herbicide are responsible in large part for toxic action, and the authors postulated some sink mechanism may have been in action to ameliorate toxicity.

### Case series

The LIPAEMIC investigators reported 48 cases of use of ILE where details of intoxicant, treatment and outcome were entered into an online registry. Complete capture of all cases treated in contributing centres was unlikely based on declining reporting rates over the time of data collection, meaning that inherent positive publication bias in case reports and series may still have been acting on this dataset. Increases were seen over a short timespan in both GCS in cases where lipid was used for altered conscious state (median GCS increasing from 4 to 8 over 30 minutes) and systolic blood pressure in shocked patients (median systolic pressure increasing from 70 to 90 mmHg over 30 minutes). One serious complication (bronchospasm) and two minor adverse effects were recorded. Given the nature of data collection, these results hold significance as contributors to hypothesis generation [55].

The ToxIC consortium reported a case series of nine uses of ILE from three tertiary referral centres [56]. Indications for use were post-cardiac arrest or refractory shock defined as circulatory compromise requiring the use of more than one vasopressor. Five of the nine patients survived, which was considered a positive outcome for ILE.

Three case series have been reported as conference abstracts. The Washington poisons centre reported five cases of use [57]: bupivacaine toxicity (asystole), and carbamazepine, hydroxychloroquine, flecainide and bupropion toxicity (seizures and hypotension). All cases were associated with clinical resolution of acute effects. Kapadia and colleagues [58] reported two cases of tricyclic antidepressant toxicity refractory to maximal therapy including alkalinisation and hypertonic saline whose clinical course improved after ILE. Jovic-Stosic and colleagues [59] reported a series of nine uses of ILE where the indication for use was poor response to vasopressors in a series of patients intoxicated with a lipophilic toxin. Increases in blood pressure and GCS, reported as occurring in all patients, were not quantified. A decrease in free verapamil concentration was described in one patient after ILE, with this same patient also exhibiting evidence of acute respiratory distress syndrome.

### Case reports

Two recent publications have summarised individual cases reported in the literature [8,10]. Case report data can be instructional in offering insights into therapies, and are particularly useful when outlining an 'all or nothing' type clinical situation. The significant limitation of such analyses is that for any effect published - both positive and negative - it is not known how many unpublished cases were treated. The absence of an accurate numerator significantly limits the generalisability of such collections of cases. With this as a major caveat, the review of Waring [10] found that there was reported improvement in 51 of 76 cases following administration of ILE to treat poisoning (28 % of reported cases being LAST, 26 % being cardiovascular drugs, 25 % psychotropic agents and 11 % antiepileptic).

## Controversies

### Porcine models in lipid resuscitation

Studies investigating ILE as antidote that report negative results in animal models have mainly used the porcine model. Swine are known to be more prone to complement-mediated allergic type reactions with liposomal formulations, and this may be important in negative studies involving ILE. Weinberg and Rubinstein [60] surveyed all the authors of papers using the porcine model for ILE/antidote research and found a majority noted skin discoloration in the ILE-treated subjects consistent with such an anaphylactoid phenomenon. Two of 10 lipid treated pigs in a protocol investigating amitriptyline distribution required adrenaline boluses, despite baseline haemodynamics having returned to normal before study treatment and end-myocardial levels of amitriptyline being lower in the lipid-treated group [36]. What data are available on ILE as antidote do not appear to indicate that such reactions have occurred in humans.

### Vasopressor coadministration with intravenous lipid emulsion in local anaesthetic systemic toxicity

Literature reports of vasopressor administration in concert with ILE-based resuscitation from local anaesthetic induced cardiac arrest in animal models have returned conflicting findings. In 2009, Hiller and colleagues [61] demonstrated that epinephrine impaired resuscitation from bupivacaine-induced asystole in rats in a dose-dependent fashion. Epinephrine injection was associated with elevated serum lactate, acidosis, and lesser haemodynamic recovery. Similarly, in earlier work Weinberg and colleagues [62] demonstrated the superiority of ILE over both epinephrine and a vasopressin/epinephrine combination [63] in rodent models of bupivacaine-induced cardiac arrest. Conversely, Mayr and colleagues [64] demonstrated superior recovery with vasopressors alone in a swine model of bupivacaine-induced cardiac arrest; albeit induction of cardiac arrest was accompanied by significant hypoxia. Mauch and colleagues [65] furthermore reported lesser recovery from bupivacaine-induced shock with ILE alone compared with adrenaline, and greater return of spontaneous circulation and survival in piglets receiving adrenaline with or without ILE in bupivacaine-induced cardiac arrest. Administration of high dose (100 mcg/kg) adrenaline in concert with ILE in bupivacaine-induced cardiac arrest has almost universally resulted in deleterious outcome in animal models. What appears clear is that avoidance of hypoxia (known to exacerbate local anaesthetic toxicity), and ensuring adequacy of cardiopulmonary resuscitation (and thereby coronary perfusion) is essential in effecting recovery. Omission of vasopressors, and adrenaline in particular, from resuscitation algorithms in LAST at this juncture remains controversial.

### Potential interaction with other resuscitation drugs

The generic nature of the sink hypothesis in ILE-based resuscitation from toxin-induced cardiac arrest has led some to question potential interaction with therapeutically administered lipophilic drugs. It would seem apparent that any effect on redistribution of toxic agents might also likewise affect agents administered therapeutically. ILE has been shown to sequester amiodarone in plasma, and ameliorate its hypotensive action when given in overdose in whole pigs [32]. The clinical effect of potential ILE-mediated pharmacokinetic modulation on therapeutically administered pharmaceuticals, however, remains uncertain. There are no direct published data on the relative roles of ILE and extracorporeal life support, with decisions to do with the use of ILE when extracorporeal life support is available in a timely fashion being necessarily based on clinical judgement.

### Enteric overdose

In keeping with the pharmacokinetic theory of ILE’s mechanism, increased gastrointestinal absorption of lipophilic intoxicant might be predicted with ILE use as blood with increased affinity for highly soluble xenobiotics perfuses intestinal mucosa. ILE administration early in the course of enteric overdose may thereby paradoxically serve to increase gut absorption and speed development of systemic toxicity. While data in support of this are still emerging [42-44], significant caution should be exercised when considering ILE administration early in the course of life-threatening enteric overdose and steps should be taken prior to use of ILE to ensure absorption is minimised.

## Safety

Administration of ILE for its antidotal effect remains an ‘off-label’ indication. Much of what is known about ILE toxicity is derived from historic studies of use in parenteral nutrition situations. When administered for this indication, allergic phenomena, pancreatitis, induced bacteraemia, fat embolism, and myocardial failure are possible. Few reports of adverse events when administered in the context of life-threatening poisoning exist, with hyperamylasaemia [66], and adult respiratory distress syndrome being reported [5]. Lipaemia-induced impairment in laboratory processing [67] may hold considerable clinical significance, particularly for patients for whom laboratory data are pertinent to management, such as alkalinised tricyclic toxic patients. Early toxicity evaluation in rodents demonstrating a median lethal dose (LD50) of 67.72 ml/kg when administered as bolus therapy seemingly provides for a wide therapeutic margin [68]. Further reporting of safety data is nevertheless required before final safety recommendations can be formalised.

## Clinical implications

The sporadic and unpredictable nature of LAST renders randomised controlled study of ILE in this setting difficult to impossible. The level of evidence available - animal models and case data - is supportive of effect and not likely to be increased. Reflecting this, ILE has been incorporated into LAST practice guidelines of governing bodies for anaesthetists worldwide [14].

Typical administration regimes recommend initial bolus injection of 1.5 ml/kg 20 % lipid emulsion followed by an infusion of 15 ml/kg/hour with accommodation for additional boluses to be given at 5 minute intervals. A recommendation not to exceed 12 ml/kg ILE has been proposed. Defining maximum length of treatment has not been explored in depth, although there is an isolated reported case where low dose lipid was used for some days [69].

Recommendation of ILE use in non-local anaesthetic drug poisoning remains controversial. While the present review outlines that the level of evidence in this area is evolving, the weight of evidence remains the same as in the field of local anaesthetics. Such a level of evidence can rationally be extended to the clinical situation of the patient *in extremus*, but extending any recommendation beyond this situation would require further human study. Furthermore, the pharmacokinetic consideration for ILE potentially exacerbating toxicity early in the course of enteric overdose necessitates proceeding with caution. ILE has been suggested as reasonable by the American College of Medical Toxicology, American Heart Association, and European Resuscitation Council for patients exhibiting intractable haemodynamic instability, or overt arrest, secondary to lipophilic drug toxicity that has proven refractory to available therapies. In this instance administration of ILE in accord with guidelines for use in LAST appears reasonable. It is the opinion of the authors that, at present, ILE utilisation for the purpose of reversal of drug-induced coma cannot be recommended outside the confines of a randomised clinical trial.

## Future research

Human randomised trials are possible in this field and necessary to evaluate whether ILE has a more general role in treatment of intoxicated patients. Registry data aimed at capture of all instances of ILE application are also required to determine an accurate denominator of ILE use, such that clinical performance characteristics may be inferred. Similarly, safety data in humans cannot be derived unless all cases of ILE use and outcomes are collated to central registries as significant adverse events may occur rarely and require many thousands of uses to identify.

Basic science experimentation is furthermore required to delineate optimal doses and formulations of ILE for human application. Research exploring the utility of liposomal detoxification vesicles has proven informative, with engineered liposomes demonstrating ability to sequester lipophilic toxins with far greater affinity than standard ILE *in vitro* [70]. Subsequent use in animal models has shown the ability of such formulations to effect meaningful recovery [71].

## Conclusion

Recent advances in understanding the mechanism of ILE treatment in poisoning suggest ion channel, metabolic and cardiotonic effects are of importance along with the more frequently quoted pharmacokinetic (sink) theory. While the evidence base for ILE use in acute drug intoxication is evolving, the present evidence supports use of ILE only in LAST and in lipophilic cardiotoxin intoxication when there is an immediate threat to life, and other therapies have proven ineffective.

## Abbreviations

GCS: Glasgow Coma Scale
ILE: Intravenous lipid emulsion
LAST: Local anaesthetic systemic toxicity
